# An Adjuvant-Free Mouse Model Using Skin Sensitization Without Tape-Stripping Followed by Oral Elicitation of Anaphylaxis: A Novel Pre-Clinical Tool for Testing Intrinsic Wheat Allergenicity

**DOI:** 10.3389/falgy.2022.926576

**Published:** 2022-06-24

**Authors:** Haoran Gao, Rick Jorgensen, Rajsri Raghunath, Perry K. W. Ng, Venu Gangur

**Affiliations:** ^1^Food Allergy and Immunology Laboratory, Department of Food Science and Human Nutrition, Michigan State University, East Lansing, MI, United States; ^2^Cereal Science Laboratory, Department of Food Science and Human Nutrition, Michigan State University, East Lansing, MI, United States

**Keywords:** wheat allergy, hypothermic shock response, systemic anaphylaxis, mouse model, cytokine

## Abstract

Wheat is a major food allergen per the regulatory bodies of various nations. Hypersensitivity reactions to wheat have been steadily increasing for reasons that are not completely understood. Wheat-allergy models typically use adjuvants to induce sensitization to wheat proteins followed by an intraperitoneal challenge to elicit anaphylaxis. Although these models are very useful, they lack the ability to reveal the intrinsic allergenicity potential of wheat. To improve the mouse model of wheat allergy, we tested the hypothesis that repeated skin application of salt-soluble protein extract (SSPE) from durum wheat will clinically sensitize the mice to oral anaphylaxis to SSPE. Balb/c mice were bred and maintained on a plant-protein-free diet and used in the experiments. Adult female mice were exposed to SSPE once a week for 9 weeks *via* a solution on intact skin. Sensitization was measured by SSPE-specific IgE (sIgE) antibody and total IgE (tIgE) levels. Oral anaphylaxis was quantified by hypothermic shock response (HSR), and mucosal mast cell response (MMCR) was quantified by measuring MMCP-1 after oral challenge. Using single mouse data, correlation analyses were performed to determine the relationship among the allergenicity readouts. Spleen cytokines were quantified using a protein microarray method. Our results show that (i) repeated skin exposures to SSPE elicited robust increases in the sIgE and tIgE levels; (ii) skin exposure to SSPE was sufficient to sensitize mice for oral anaphylaxis and MMCR; (iii) both HSR and MMCR showed a strong correlation with each other, as well as with sIgE, and a modest correlation with tIgE levels; (iv) selected Th2/Th17/Th1 cytokines were elevated in skin-sensitized mice; and (v) oral allergen-challenged mice showed selective elevation of IL-6 and a panel of chemokines compared to saline-challenged mice. Together, we report the development and characterization of a novel adjuvant-free wheat-allergy mouse model that uses skin sensitization without tape-stripping followed by oral elicitation of anaphylaxis. Furthermore, validation of quantifiable wheat allergenicity readouts makes this model particularly suitable as a pre-clinical testing tool to assess the intrinsic sensitization/oral-anaphylaxis elicitation potential of novel wheat proteins (e.g., processed wheat) and to develop hypo/non-allergenic wheat products.

## Introduction

Food allergies are chronic potentially fatal reactions to common food proteins mediated by the immune system (1). They are on the rise for reasons that are not well understood at present (2). In the United States, their prevalence is 8% among children and 10.8% among adults (3, 4). Other countries, such as Canada, EU, Japan, and Australia, have reported a similar trend (5–8). One decade ago, the estimated annual economic impact of food allergies in the United States was $24.8 billion; the current updated impact information is unavailable (9). In addition to its increasing prevalence, emergency department visits due to food-induced anaphylaxis are also on the rise (4, 10). There is no cure for food allergy at present (2). It is reported that the quality of life of food-allergic individuals is significantly impaired as constantly paying stringent attention to diet can introduce extra burdens on individuals, as well as on their families, schools, and healthcare takers (11).

Wheat is the world's second most-produced cereal, after corn (12). However, per capita consumption of wheat flour has been decreasing in the last two decades in the United States by ~ 7.7% (from 144 lbs./person in 1999 to 131 lbs./person in 2019) (13). Wheat is among the 8–14 major allergenic foods that are regulated by multiple countries, including the United States, Canada, EU, United Kingdom, Australia, and New Zealand (12, 14–19). Besides wheat, other major food allergens are milk, fish, shellfish, peanuts, tree nuts, eggs, soybean, sesame, celery, lupin, mustard, and sulfites (16, 20, 21).

In general, wheat has been reported to cause two distinct types of immune-mediated adverse reactions: (i) IgE antibody-mediated allergic/anaphylactic reactions, which are potentially deadly; and (ii) non-IgE-mediated reactions that tend to be chronic conditions; these include autoimmune celiac disease, non-celiac gluten sensitivity, and eosinophil-mediated inflammatory gut reactions (i.e., eosinophilic esophagitis and eosinophilic gastritis) (22–24). Validated mouse models play a critical role in advancing the knowledge of the mechanisms underlying these diseases so that novel methods of prevention and treatment can become available.

The prevalence of wheat allergy in the United States among adults is 0.9%−3.6% (25, 26). Its prevalence among United States children is 0.2%−1.3% (27–29). In both Europe and Australia, the prevalence of wheat allergies among adults and children is 0.4% and 1%, respectively (30–32). The seriousness of wheat allergy is illustrated by the reports that more than half of the affected children have experienced anaphylactic reactions, which can be potentially fatal (33, 34). Although 65% of children with wheat allergy outgrow it, a significant proportion (35%) continue to have persistent wheat allergy into adulthood with a continued risk of life-threatening reactions for the rest of their lives (35). Even so, wheat allergens are under-researched relative to other allergenic foods, such as peanuts, tree nuts, milk, and egg. For example, an adjuvant-free mouse model to study oral elicitation of anaphylaxis to wheat is unavailable at present–the focus of this study.

Wheat is a highly nutritious staple food, particularly because of its high protein content. On a dry weight basis, wheat contains 10%−14% of protein that includes gluten and non-gluten fractions. The non-gluten fraction (i.e., albumins and globulins; water/salt-soluble proteins) accounts for 15%−20% of the total proteins. The remaining 80%−85% of total protein is comprised of glutens (that include gliadins and glutenins) (36). Although wheat proteins are important sources of nutrients for most people, both types of proteins are also equally important sources of allergens for wheat-allergic subjects. Relative to non-glutens, most published research on wheat proteins has focused on glutens, and therefore, there is a need to advance the knowledge on the allergenicity of non-gluten proteins also—the focus of this study.

Animal models are critical to advancing our knowledge of wheat food allergies (36). Consequently, dogs, rats, and mice have been used to develop wheat-allergy models (37–50). Mice are very attractive and popular due to relatively lower costs, wide availability of immunological reagents for mouse protein targets, and availability of gene knockout strains (36). Among the mouse strains, Balb/c was shown to exhibit wheat allergenicity similar to that of humans (43). However, there are two major limitations facing the wheat allergenicity mouse models: (i) adjuvants for inducing systemic sensitization or tape-stripping of stratum corneum of skin for skin sensitization are commonly used that tend to elevate subject sensitivity; this however limits the ability to assess the intrinsic allergenicity potential of any tested wheat proteins, including novel proteins, such as processed wheat proteins and novel wheat varieties/lines; and (ii) intraperitoneal injection to elicit anaphylaxis does not simulate the oral-wheat-induced anaphylaxis noted in humans (48). An adjuvant-free mouse model without tape-stripping on the other hand is more desirable as it makes data interpretation of the intrinsic allergenicity of wheat proteins possible, and therefore, is more suitable for allergenicity testing (43). Furthermore, oral elicitation of anaphylaxis using wheat proteins will enable studying mechanisms of oral anaphylaxis and assist in developing novel methods to prevent and treat oral anaphylaxis. Therefore, an adjuvant-free skin-sensitization/oral-anaphylaxis-elicitation mouse model of wheat allergenicity is urgently needed—the focus of this study.

Here, we have tested the hypothesis that repeated skin application of salt-soluble protein extract (SSPE) from durum wheat will clinically sensitize the mice for oral anaphylaxis to those wheat proteins. There were seven objectives for this study: (i) to establish a colony of plant-protein-free Balb/c mice; (ii) to test for specific (s)IgE and total (t)IgE antibody response to SSPE from durum wheat upon repeated skin exposures to SSPE in adult female mice; (iii) to test for oral anaphylaxis to SSPE as quantified by hypothermic shock response (HSR) in skin-sensitized mice; (iv) to evaluate mucosal mast cell response (MMCR) upon oral challenge with SSPE in skin-sensitized mice; (v) to determine the correlations among four quantifiable readouts of wheat allergenicity (sIgE, tIgE, HSR, and MMCR); (vi) to evaluate spleen cytokine response in skin-sensitized mice; and (vii) to identify the spleen immune markers that are elevated upon oral SSPE vs. saline challenge in SSPE-allergic mice.

Together, we report the development and characterization of a novel wheat-allergy mouse model that uses skin sensitization without tape-stripping, followed by oral elicitation of anaphylaxis. Furthermore, validation of quantifiable wheat allergenicity readouts makes this model particularly suitable as a pre-clinical testing tool to assess the intrinsic sensitization and oral-anaphylaxis-elicitation potential of wheat proteins, including novel wheat proteins (e.g., processed wheat), and in the development of hypo/non-allergenic wheat proteins.

## Materials and Methods

### Chemicals and Reagents

Biotin-conjugated rat anti-mouse IgE-paired antibodies were purchased from BD BioSciences (San Jose, CA, United States). *p*-Nitro-phenyl phosphate was obtained from Sigma (St Louis, MO, United States). Streptavidin alkaline phosphatase was obtained from Jackson ImmunoResearch (West Grove, PA, United States). BSA standard (at 2 mg/ml) was purchased from Sigma (St. Louis, MO, United States). Alkaline copper tartrate was purchased from BioRad (Hercules, CA, United States). Folin reagent was purchased from BioRad (Hercules, CA). The following reagents were obtained as listed: IgE Mouse Uncoated ELISA Kit with Plates; Streptavidin-HRP, TMB substrate; MCPT-1 (mMCP-1) Mouse Uncoated ELISA Kit with Plates; Avidin-HRP, TMB substrate (all from Invitrogen, MA, United States); Tissue Protein Extraction Reagent (T-PER™, a proprietary detergent in 25 mM bicine, 150 mM sodium chloride, pH 7.6; from ThermoFisher Scientific, MA, United States); protease (serine, cysteine, and acid proteases, and aminopeptidases) inhibitor cocktail (Sigma-Aldrich, MO, United States).

### Mice Breeding and Establishment of a Plant-Protein-Free Mouse Colony

Adult Balb/cJ breeding pairs were purchased from The Jackson Laboratory (Bar Harbor, ME). Upon arrival, they were placed on a plant-protein-free diet (AIN- 93G, Envigo, Madison, MI). After acclimating for a week, the breeding pairs were set up as one male:two females per cage. Pregnant females were separated and after delivery, pups were weaned at 4 weeks. Adult female mice (6–8 weeks) were used in the experiments. All mice were maintained on the plant-protein-free diet (AIN- 93G) throughout the study. All animal procedures were as per the Michigan State University policies.

### Preparation of Salt-Soluble Protein Extract From Durum Wheat Flour

Durum wheat flour (genomes AABB, variety Carpio) was used for protein extraction. Salt-soluble protein extract (SSPE) was prepared using a method published previously (51). Briefly, flour and sterile 0.5 M NaCl were mixed in a 1:10 ratio (m/v), and stirred continuously for 2 h followed by centrifugation (5,000 × g, 10 min) at 20°C. The supernatant was frozen at −70°C overnight and freeze-dried the next day. Lyophilized SSPE powder was reconstituted with sterile saline. Protein concentration was determined using the Bio-Rad method (52).

### Skin Sensitization, Bleeding, and Plasma Sample Preparation

Adult mice were used in the experiments (specific numbers for each experiment are provided in the RESULTS Section). Hair on the rump of the mice was removed using a hair clipper (Philips, Amsterdam, the Netherlands). A total of 50 μl of durum wheat SSPE (10 mg/ml) or vehicle (10% sterile NaCl solution) was applied over both sides of the clipped area on the rump (1 mg in 100 μl per mouse per exposure). The mice were then covered with a non-latex bandage (Johnson & Johnson, New Brunswick, New Jersey) for 1 day. The above procedure was repeated weekly for 9 weeks. Bleeding was done 1 week before the 1st exposure and after the 8th exposure *via* the saphenous vein. Blood was collected in anticoagulant (lithium heparin) coated vials (Sarstedt Inc MicrovetteCB 300 LH, Germany) and centrifuged to harvest the plasma. Individual plasma samples were stored at −70°C until used in the analysis.

### Elicitation of Oral Anaphylaxis and Hypothermic Shock Responses

Two weeks after the 8th exposure to durum SSPE, the mice were challenged orally with durum wheat SSPE (20 mg per mouse in 300 μl sterile saline) or vehicle (300 μl sterile saline) by using curved feeding needles (22-gauge, length: 1.4 in, Kent Scientific, Torrington, CT, United States). Specific number of mice for each experiment is provided in the RESULT Section. Rectal temperature (°C) was recorded before and after the challenge every 5 min up to 30 min by using a thermometer with a probe (DIGI-SENSE, MA, United States). Actual temperatures and changes in rectal temperature (Δ°C) every 5 min compared to the pre-temperatures for each mouse were used in the analyses.

### Measurement of Wheat SSPE-Specific IgE Antibody Levels

Wheat SSPE-specific(s) IgE antibody levels in the plasma were measured using an ultrasensitive ELISA method as we have reported before (47, 48, 53). This method was a modified version of the published method we have reported previously for food-specific IgE antibody measurement in the mouse system (54). Briefly, 96-well plates (Corning 3369) were coated with durum SSPE, followed by blocking with 5% gelatin, washing, plasma sample addition, washing, addition of biotin-conjugated anti-mouse IgE antibody, washing, and the addition of Streptavidin Alkaline Phosphatase and PNPP detection system as described previously (47, 53). Individual mouse samples were tested in quadruplicate.

### Measurement of Total Plasma IgE Concentration

Total(t) IgE concentrations were determined using a commercial ELISA kit (Invitrogen, Waltham, MA) that contained antibody pairs (i.e., anti-mouse IgE as capture antibody and biotin-conjugated anti-mouse IgE as detection antibody) and a recombinant mouse IgE standard as described before (47, 48). Briefly, 96-well plates (Corning Costar 9018) were coated with capture antibody (anti-mouse IgE), followed by adding samples and standards (recombinant mouse IgE). A secondary antibody (biotin-conjugated anti-mouse IgE) was then added. Detection was based on Streptavidin-HRP and TMB substrate system. Assay sensitivity: 4 ng/ml. The standard range used for quantification: 250 – 4 ng/ml. Individual mouse samples were tested in quadruplicate.

### Measurement of Wheat SSPE-Specific IgG1 Antibody Levels

Wheat SSPE-specific(s) IgG1 antibody levels in the plasma were measured using an ELISA method as we have reported before (47, 48, 53). Briefly, 96-well plates (Corning 3369) were coated with durum SSPE, followed by blocking, sample addition, and secondary antibody (biotin-conjugated anti-mouse IgG1 antibody). The plate was developed by Streptavidin Alkaline Phosphatase and PNPP detection system as described previously (47, 53).

### Quantification of Mucosal Mast Cell Protease-1 (MMCP-1) Level

The blood collected at 1-h post-challenge was used in measuring MMCP-1 levels (ng/ml) in the plasma using an ELISA-based method according to Invitrogen as we described previously (47, 48). Briefly, 96-well plates (Corning Costar 9018) were coated with capture antibody (anti-mouse MMCP-1), followed by adding samples and standards (recombinant mouse MMCP-1). A sandwich was then formed when a secondary antibody (biotin-conjugated anti-mouse MMCP-1) was added. Detection was based on avidin-HRP and TMB substrate system. Assay sensitivity: 120 pg/ml. The standard range used for quantification: 15,000–120 pg/ml. Individual mouse samples were tested in quadruplicate.

### Preparation of Spleen Extract and Analysis of Immune Markers

The mice were euthanized 1 h after the oral challenge. Their spleens were harvested, snap-frozen, and stored at −70°C until used for tissue extraction, which was performed using the method we have described before (48). Briefly, spleen tissues were immersed in a Tissue Protein Extraction Reagent (T-PER™) with a protease inhibitor. For each 100 mg of tissue, 10 μl of protease inhibitor per 1 ml T-PER buffer was used. The spleen tissue was homogenized by ultra-sonication for 30 s twice, with a 5-min interval in between, and then, was rested for 15 min before centrifugation (13,500 × g) for 10 min at 4°C. The supernatants were collected and stored in aliquots at −70°C until used in the analysis. The Quantibody microarray (RayBiotech, Atlanta, GA) was used to quantify a panel of immune biomarkers. All samples were analyzed in quadruplicate using standards (https://www.raybiotech.com/mouse-cytokine-array-q2000/).

### Statistics

An online software service was used in these analyses (https://www.socscistatistics.com/tests/). The significance level was set at *p* < 0.05. Student's *t*-test was used to compare the two groups and ANOVA was used for multiple comparisons. Pearson correlation analysis was used to determine the relationship among the allergenicity readouts.

## Results

### Repeated Skin Exposures to Durum Wheat SSPE Elicits Robust Specific-IgE and Specific-IgG1 Antibody Responses in Balb/c Female Mice

**Figure 1:**
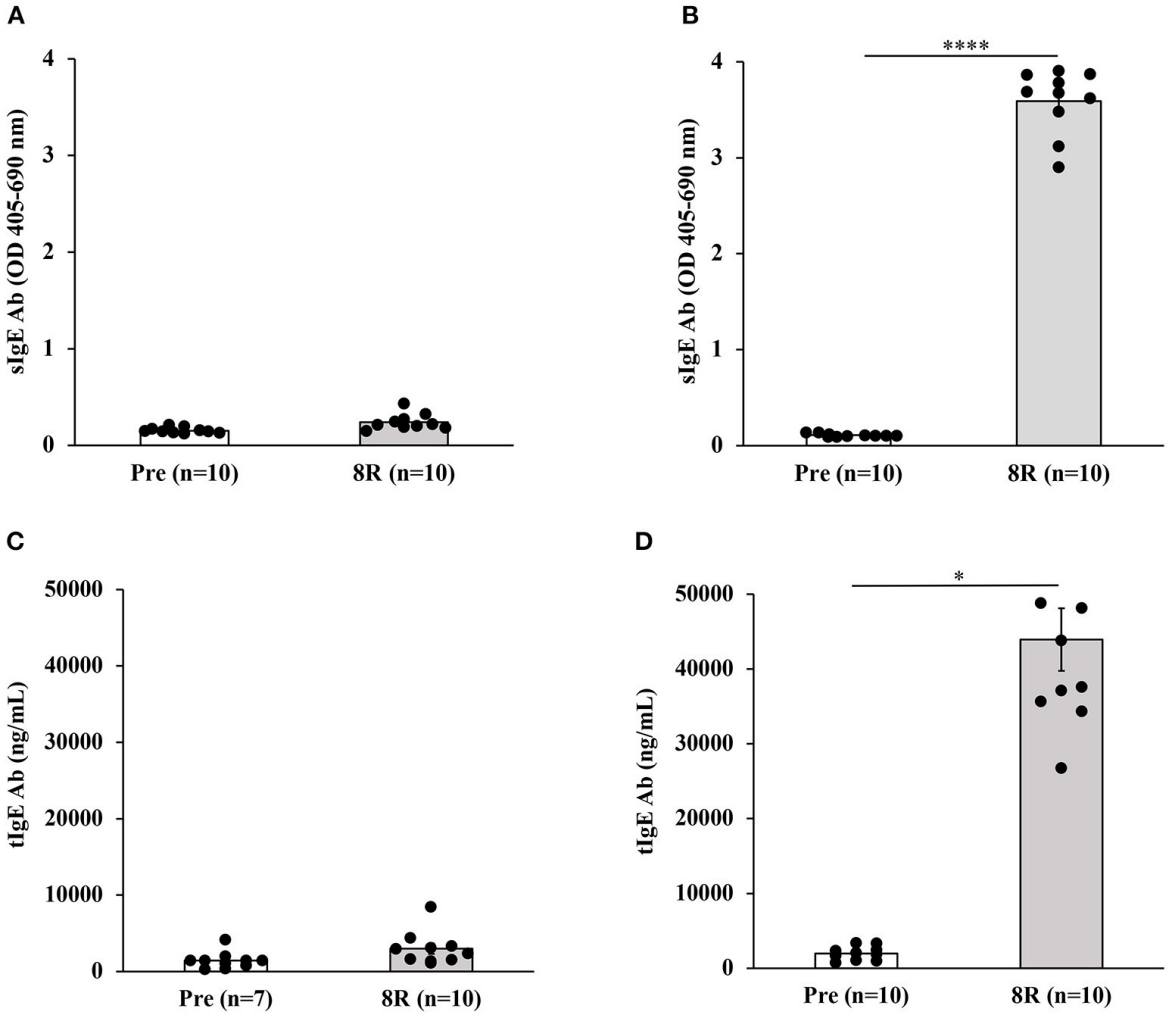
Transdermal exposure to durum wheat (genomes AABB) SSPE elicited exposure-dependent SSPE-specific (s) IgE antibody responses and elevation of total (t) IgE in Balb/c mice that correlate with each other. Mice were exposed to SSPE or to saline as described in Materials and Methods. Blood was collected before 1st exposure (Pre) and after 8th exposure (8R). Plasma was used in measurement of SSPE-specific IgE levels (OD 405–690 nm) using an ELISA method described previously. **(A)** SSPE-specific IgE levels in control mice. **(B)** SSPE-specific IgE levels in sensitized mice. ****p < 0.001; Ab, antibody. **(C)** Total IgE levels in control mice. **(D)** Total IgE levels in sensitized mice. *p < 0.05.

Groups of Balb/c female mice were transdermally exposed to durum wheat SSPE or saline by repeated weekly exposures as described in the Methods. The SSPE used in this study had been characterized for protein quantity and quality previously (Supplementary Figure 1). Blood collected before vs. after 8 skin exposures was used in measurements of sIgE levels. As can be seen in Figures 1A,B, a robust induction of sIgE antibody levels after transdermal exposure with SSPE but not vehicle was noted (~17-fold increase in allergic mice vs. vehicle control mice). We noted that robust sIgE responses were elicited even after four skin exposures in this model (Supplementary Figure 2). We also noted specific (s)IgG1 antibody responses upon skin exposures to SSPE (Supplementary Figures 3A,B).

### Repeated Skin Exposures to Durum Wheat SSPE Also Elevates Total IgE Levels in Balb/c Mice

We measured the tIgE concentrations in the plasma before and after skin sensitization. As evident, a dramatic increase in tIgE levels was observed in SSPE-sensitized but not in vehicle-sensitized mice (~50-fold increase in allergic mice vs. vehicle control mice; Figures 1C,D).

### Repeated Skin Exposures to Durum Wheat SSPE Is Sufficient to Clinically Sensitize Mice for Anaphylactic Responses After Oral Allergen Challenge

**Figure 2:**
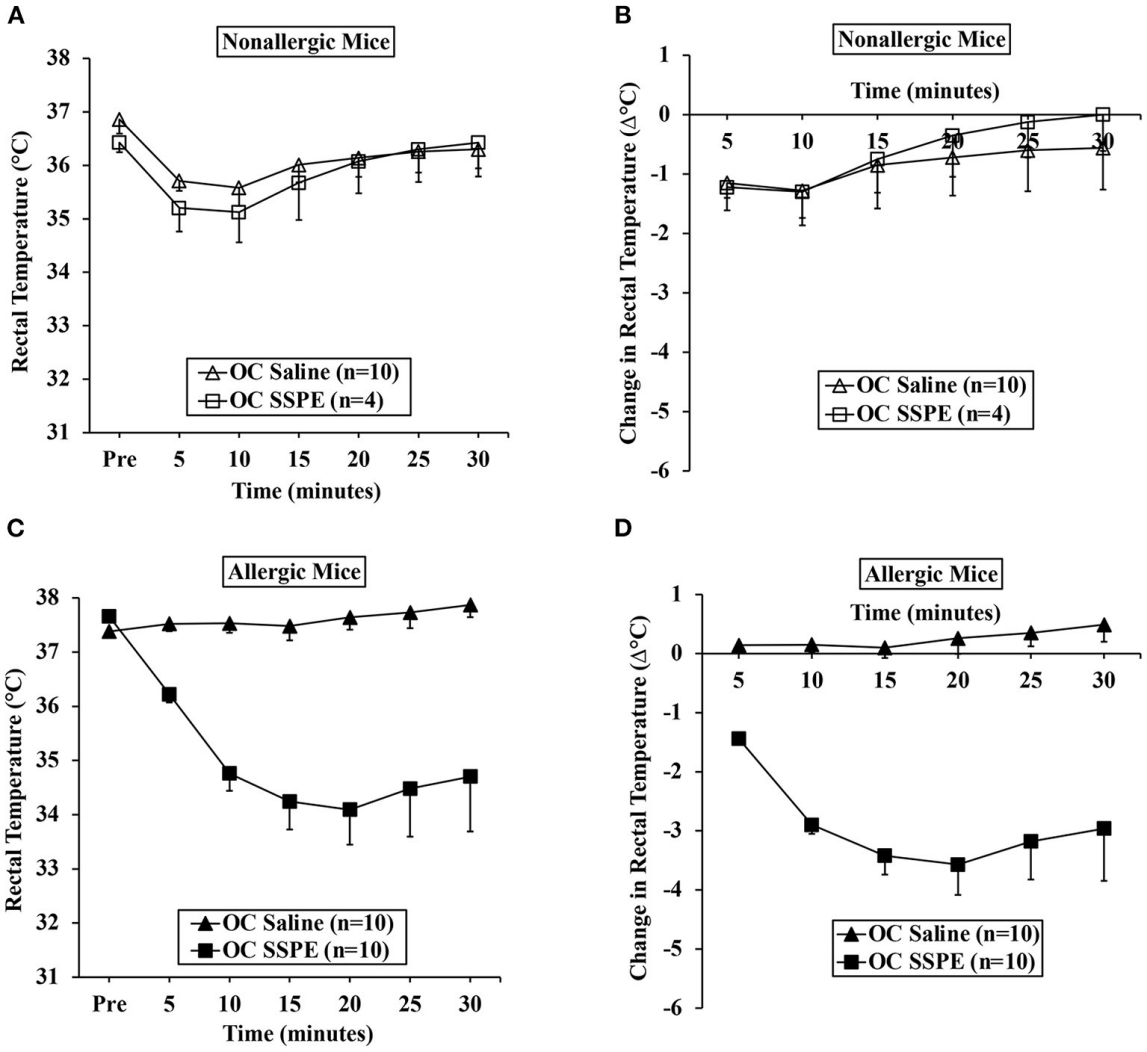
Transdermal exposure to durum wheat (genomes AABB) SSPE sensitized Balb/c mice for anaphylaxis upon oral challenge. Mice exposed to SSPE or to saline were orally challenged (OC) as described in MATERIALS AND METHODS. **(A)** Rectal temperatures (°C) at indicated time points in non-allergic mice challenged with SSPE or saline. **(B)** Change in rectal temperature (Δ°C) at indicated time points in non-allergic mice challenged with SSPE or saline. **(C)** Rectal temperatures (°C) at indicated time points in allergic mice challenged with SSPE or saline. **(D)** Change in rectal temperature (Δ°C) at indicated time points in allergic mice challenged with SSPE or saline.

We used parallel groups of skin-sensitized mice to induce anaphylaxis by performing the allergen challenge *via* the oral route. Anaphylactic reactions were quantified by hypothermic shock reactions (HSR) using rectal thermometry. The actual temperatures before and after oral challenge with the allergen (15 mg/mouse) or saline at 5-min intervals are shown in Figure 2A. The change in temperature every 5 min post-challenge compared to pre-challenge temperatures is shown in Figure 2B. There was no HSR upon vehicle (i.e., zero allergen) challenge. Furthermore, oral administration of allergen to non-allergic mice also did not elicit HSRs. On the contrary, acute HSRs were observed upon oral allergen challenge in skin-sensitized mice (Figures 2C,D). Significant HSRs were noted from 15 to 30 min (ANOVA, *p* < 0.05).

### Hypothermic Shock Responses of Durum Wheat SSPE-Sensitized Allergic Mice Correlate More Strongly With Specific-IgE Antibody Than the Total IgE Levels

**Figure 3:**
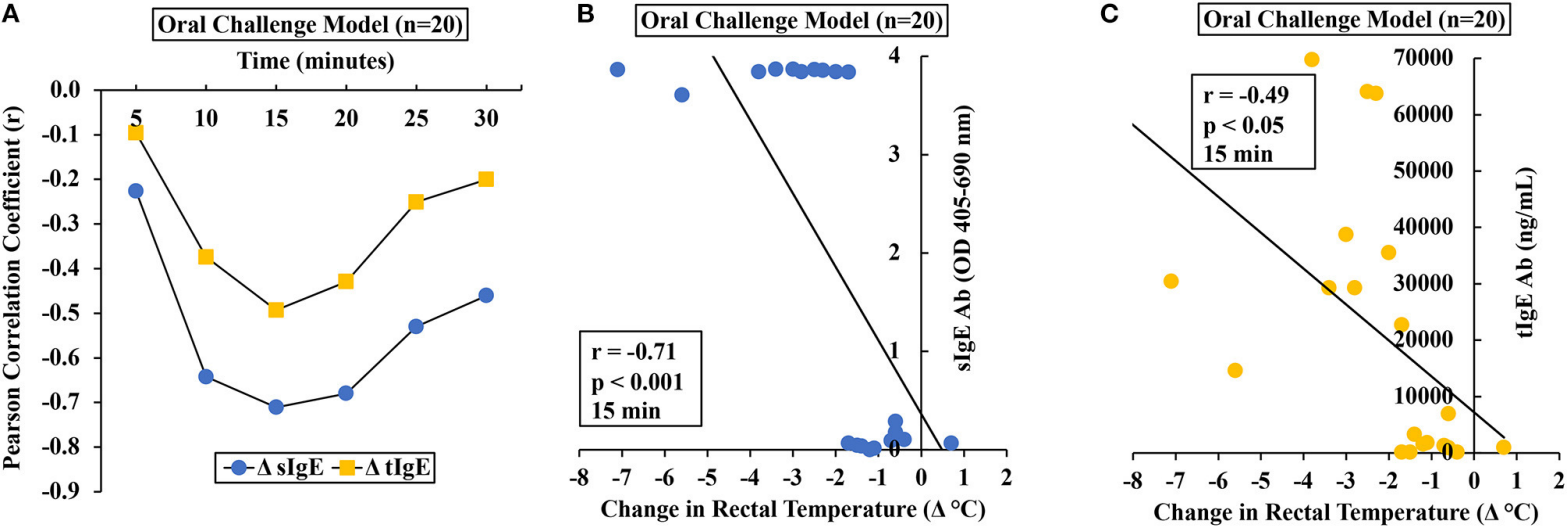
Anaphylactic responses in allergic mice upon oral challenge with durum wheat (genomes AABB) SSPE correlated with specific and total IgE levels. Mice were exposed to SSPE or to saline and challenged as described in Materials and Methods. **(A**–**C)** Pearson correlation coefficient (*r*) between antibody responses (sIgE or tIgE levels) and change in rectal temperature (Δ°C) in mice sensitized and orally challenged with SSPE (20 mg). **(A)** Pearson correlation coefficient (*r*) at indicated time points. **(B)** Pearson correlation analysis between sIgE and Δ°C at 15 min post challenge. **(C)** Pearson correlation analysis between tIgE and Δ°C at 15 min post challenge. sIgE, specific IgE; tIgE, total IgE; Ab, antibody.

Using single mouse data, we determined the relationship between HSR and IgE antibody levels (sIgE and tIgE) in oral allergen-challenged mice. The correlation between change in the temperature (Δ°C) at every 5 min post-challenge compared to the pre-temperature and sIgE is shown in Figure 3A. As evident, both sIgE and tIgE showed significant correlations with Δ°C; however, the sIgE levels consistently showed stronger correlations with Δ°C than did the tIgE levels (Figure 3A). We noted the strong correlations at 15 min post-oral allergen challenge for both sIgE and tIgE (Figures 3B,C).

### Analysis of Mucosal Mast Cell Degranulation Responses Upon Allergen Challenge in Durum Wheat SSPE-Sensitized Mice

It has been shown in a previous study that degranulation of mucosal mast cells resulting in acute elevation of blood levels of murine mucosal cell protease (MMCP)-1 after allergen challenge is a biomarker of IgE-mediated systemic anaphylaxis in mice (55). Therefore, we determined MMCP-1 responses in allergic mice upon oral allergen challenges.

**Figure 4:**
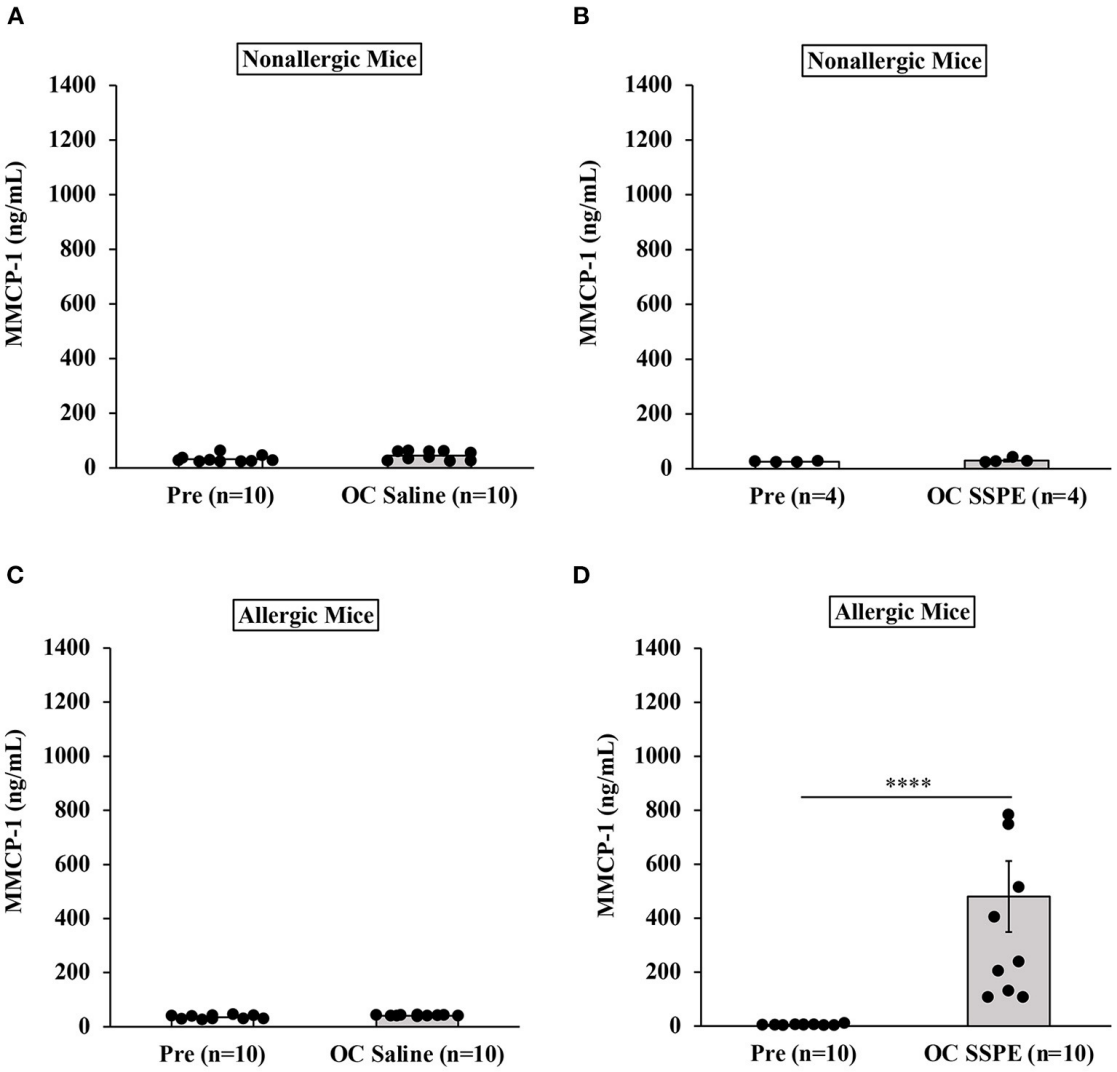
Transdermally sensitized allergic mice exhibited degranulation of mucosal mast cells upon oral challenge with durum wheat (genomes AABB) SSPE. Mice were treated as described in MATERIALS AND METHODS. Their serum mucosal mast cell protease-1 levels (ng/mL) were measured using an ELISA-based method described in the texts. **(A)** MMCP-1 levels in control mice challenged with saline. **(B)** MMCP-1 levels in control mice challenged with durum SSPE. **(C)** MMCP-1 levels in allergic mice challenged with saline. **(D)** MMCP-1 levels in allergic mice challenged with durum SSPE. ****p < 0.001. MMCP-1, mucosal mast cell protease-1.

Results of MMCP-1 responses in oral allergen-challenged mice are shown in Figure 4. As expected, non-allergic control mice did not show marked elevation of MMCP-1 levels upon vehicle oral challenges (Figures 4A,B). In contrast, allergic mice exhibited robust MMCP-1 responses upon oral allergen challenge (20 mg dose; Figures 4C,D).

### Correlation Analysis Between Mucosal Mast Cell Responses and Hypothermic Shock Responses in This Mouse Model

**Figure 5:**
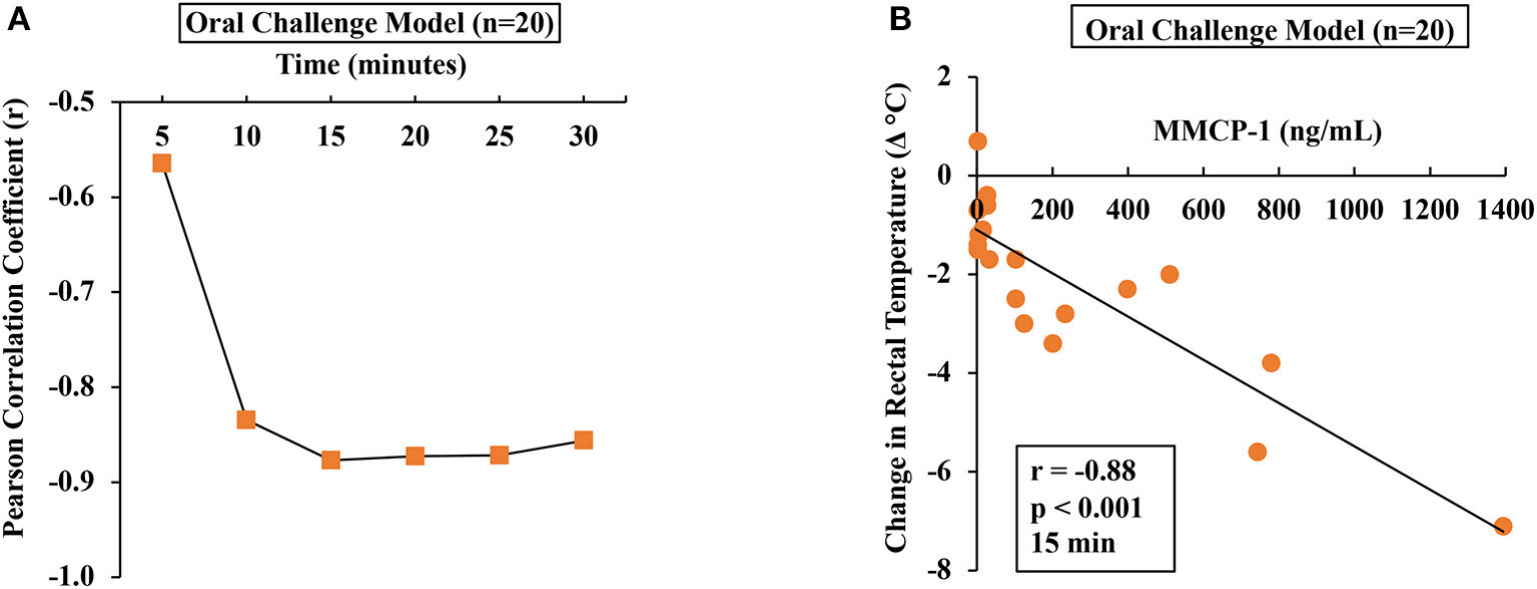
Hypothermic shock responses in durum wheat (AABB genome) SSPE-allergic mice correlated with their degranulation of mucosal mast cells. Mice received treatments as described in Materials and Methods. Their serum MMCP-1 levels (ng/ml) were compared with change in rectal temperature (Δ°C) post-challenge using Pearson correlation analysis. **(A)** Pearson correlation coefficient (*r*) at indicated time points in orally challenged allergic mice with SSPE (20 mg). **(B)** Pearson correlation analysis at 15 min post-challenge in saline-challenged control mice (*n* = 10) and SSPE-challenged (20 mg) allergic mice (*n* = 10) *via* the oral route. MMCP-1, mucosal mast cell protease-1.

To determine the relationship between HSR and mucosal mast cell responses (MMCRs), we conducted Pearson correlation coefficient analysis using single-mouse data oral allergen-challenged mice. Results of correlation analysis between HSR and MMCR in oral-challenged mice are shown in Figure 5. We observed a strong correlation (*r* = −0.88) between MMCR and HSR from 15 to 30 min post-challenge period (Figures 5A,B).

### Correlation Analysis Between Mucosal Mast Cell Responses and IgE Levels in This Mouse Model

**Figure 6:**
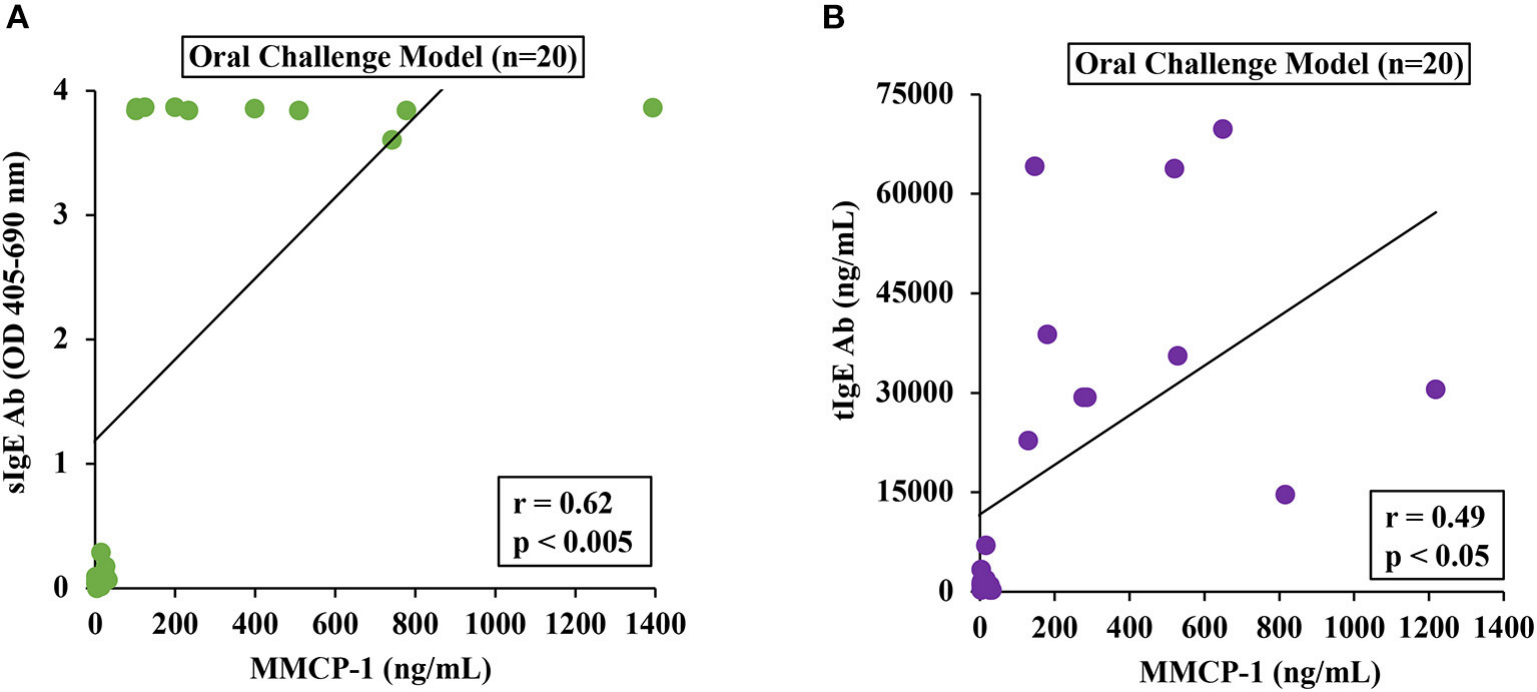
Mucosal mast cell responses upon challenge with durum SSPE correlated with specific and total IgE levels. Mice were treated as described in MATERIALS AND METHODS. Pearson correlation analysis was used to test the relationship between MMCP-1 levels (ng/ml) and antibody responses (sIgE or tIgE) in the plasma after 8th transdermal exposure (8R) to SSPE. Data from saline challenged control mice (*n* = 10) and SSPE-challenged (20 mg) allergic mice (*n* = 10) *via* the oral route. **(A)** Pearson correlation analysis between sIgE and MMCP-1 levels in orally challenged (OC) mice. **(B)** Pearson correlation analysis between tIgE and MMCP-1 levels in orally challenged (OC) mice. sIgE, specific IgE; tIgE, total IgE; Ab, antibody.

To determine the relationship between MMCRs and IgE levels, we conducted Pearson correlation coefficient analysis using single-mouse data from oral-challenged mice. We found that only sIgE showed a strong correlation with MMCR (Figures 6A,B).

### Identification of Immune Biomarkers Associated With Sensitization vs. Oral Anaphylaxis to Durum Wheat SSPE

We screened a selected panel of spleen immune makers to study the cytokine responses upon sensitization. As shown in Table 1, compared to unsensitized mice, durum wheat SSPE-sensitized mice had significantly higher levels of prototypic Th2 cytokines (IL-4, IL-5) and a Th17 (IL-17E) cytokine. Also, Th1 cytokines (IFN-γ and IL-12p70) were elevated. We also studied the spleen biomarkers that increase upon oral allergen vs. saline challenge in skin-sensitized mice. Interestingly, none of the prototypic Th2, Th17, or Th1 were significantly changed after the oral allergen challenge compared to the oral saline challenge (data not shown). However, IL-6 was the only cytokine that was significantly elevated (sensitized mice, orally challenged with saline: 36.41 ± 2.67 ng/ml; sensitized mice, orally challenged with allergen: 85.06 ± 10.57 ng/ml; student's *t*-test, two-tailed, *p* < 0.005). Furthermore, we identified the following 4 chemokines that were also significantly elevated upon oral allergen but not the saline challenge of skin-sensitized mice: CCL5, CCL20, CCL22, and CXCL1 (Table 2).

**Table 1:** Spleen cytokine levels in unsensitized mice vs. durum wheat sensitized mice.

Cytokines*	**Unsensitized mice** **(*n* = 5)**	**Sensitized mice** **(*n* = 5)**	**Student's *t*-test,** **(2-tailed)**
***IFN-γ***	<8 (LOD)	42.50 ± 9.49	*p* <0.05
***IL-2***	182.42 ± 67.46	582.19 ± 110.21	*p* <0.05
***IL-12p40***	37.43 ± 6.01	29.90 ± 6.13	ns
***IL-12p70***	18.79 ± 3.55	118.14 ± 11.81	*p* <0.001
***IL-23***	2,166.99 ± 304.05	2,547.63 ± 275.73	ns
***TNF-α***	18.23 ± 9.80	33.34 ± 17.62	ns
***IL-4***	2.37 ± 0.29	6.34 ± 1.24	*p* <0.05
***IL-5***	28.87 ± 6.27	98.36 ± 15.26	*p* <0.01
***IL-6***	7.29 ± 0.87	35.52 ± 2.60	*p* <0.001
***IL-13***	<12.1 (LOD)	<12.1 (LOD)	na
***IL-17A***	<5.9 (LOD)	<5.9 (LOD)	na
***IL-17E***	53.81 ± 10.23	181.70 ± 34.77	*p* <0.05

* *pg/mL of spleen protein extract*.

*LOD, limit of detection; ns, not significant; na, not applicable*.

**Table 2:** Identification of spleen chemokines that are increased upon oral allergen but not saline challenge in durum wheat sensitized mice.

Spleen immune markers*	**Sensitized mice orally challenged with saline (*n* = 5)**	**Sensitized mice orally challenged with allergen (*n* = 5)**	**Student's *t*-test (2-tailed)**
CCL1 (TCA3)	99.11 ± 10.16	92.97 ± 10.88	ns
CCL2 (MCP-1)	175.03 ± 48.81	111.25 ± 36.91	ns
CCL3 (MIP-1a)	<5.5 (LOD)	10.54 ± 5.04	ns
CCL5 (RANTES)	885.30 ± 17.06	951.05 ± 18.65	*p* <0.05
CCL9 (MIP-1g)	713.60 ± 7.05	740.15 ± 19.16	ns
CCL12 (MCP-5)	<2 (LOD)	3.65 ± 1.65	ns
CCL19 (MIP-3b)	57.78 ± 5.12	60.22 ± 2.52	ns
CCL20 (MIP-3a)	17.96 ± 6.45	52.75 ± 5.42	*p* <0.01
CCL22 (MDC)	214.13 ± 12.05	283.63 ± 5.00	*p* <0.005
CCL24 (Eotaxin-2)	1,045.27 ± 35.93	992.89 ± 10.24	ns
CXCL1 (KC)	50.90 ± 0.85	78.65 ± 7.70	*p* <0.05
CXCL4 (PF-4)	73,323.82 ± 1,479.44	72,812.41 ± 1,400.74	ns
CXCL11 (I-TAC)	<40 (LOD)	<40 (LOD)	na

* *pg/mL of spleen protein extract*.

*LOD, limit of detection; ns, not significant; na, not applicable*.

## Discussion

Here we tested the overall hypothesis that repeated exposure to salt-soluble protein extract (SSPE) from durum wheat *via* the skin without tape-stripping or the use of an adjuvant will be sufficient to clinically sensitize mice for oral allergen-induced anaphylaxis. Our data together support this hypothesis.

There are seven novel findings from our studies: (i) repeated skin exposures**—**once a week for 8 weeks to durum wheat SSPE dramatically elevated the sIgE levels, as well as the tIgE levels in blood; (ii) repeated skin exposures to durum SSPE is sufficient to clinically sensitize mice for oral SSPE-induced anaphylaxis as quantified by hypothermic shock responses (HSR); (iii) strong correlations between sIgE and HSR in individual mice analysis confirm that IgE antibodies contribute to the HSR; (iv) oral SSPE-induced anaphylaxis is associated with significant mucosal mast cell degranulation response (MMCR) confirming that IgE/mast cell pathway is engaged in this model; (v) significant correlations between HSR and MMCR in single mice analysis confirm that MMCR contributes to HSR; (vi) strong correlations between sIgE and MMCR in single mice analysis confirm that MMCR is mediated by the sIgE-oral SSPE interaction resulting in mucosal mast cell response in the gut; and (vii) identification of a panel of spleen immune markers that are significantly elevated upon oral allergen but not saline challenge in wheat-sensitized mice.

Wheat contains two families of allergenic proteins—gluten and non-gluten proteins. More than 100 specific allergens within these two families have been well characterized (56). Although both types of allergens can cause human wheat allergies, most mouse model studies to date have been done using gluten allergens (40–50). To advance the knowledge on the biology of non-gluten allergens, we have been characterizing the immune response to SSPE using durum wheat as a model tetraploid (genomes AABB) wheat. Using a popular and widely used alum-adjuvant-based model, we have previously demonstrated that durum SSPE when administered by intraperitoneal (IP) injections along with alum induces IgE responses and sensitizes mice for anaphylaxis upon IP injection with SSPE alone (47). Furthermore, a long-term study suggested that some of the SSPE-allergic mice developed severe atopic dermatitis (47). Later, we demonstrated that the durum SSPE can also elicit IgE responses upon skin exposure (once a week for 6 weeks) and sensitize mice for anaphylaxis upon IP injection with SSPE (48). However, it was unknown whether repeated skin exposures to SSPE from durum wheat will be sufficient to clinically sensitize mice for oral SSPE-induced anaphylaxis. Here, we demonstrate that repeated nine skin exposures (once a week for 9 weeks) clinically sensitizes mice for oral SSPE-induced anaphylaxis. Thus, together with this study, we have further advanced the scientific knowledge of allergic immune responses to durum SSPE.

Human wheat allergies develop *via* unknown mechanisms of allergic sensitization to wheat proteins. For example, skin exposure to wheat proteins, such as SSPE *via* the skin, can happen when wheat flour/dough is handled with bare hands (e.g., kitchen, baking industries). However, whether such exposures can have any clinical consequences is completely unknown. Recently, there is growing interest in studying immune responses *via* the skin environment (57, 58). Therefore, to advance the knowledge of the clinical consequence of skin exposure to food proteins, we have been studying immune responses to various types of food allergenic proteins, including tree nuts, shellfish, and sesame (59–63). Here, we further advance the biology of immune responses *via* skin without tape-stripping of stratus corneum using durum wheat SSPE and demonstrate that it can clinically sensitize Balb/c mice for oral anaphylaxis *via* the IgE/mucosal mast cell degranulation responses for the first time.

The model that we have developed and characterized in this study stands out as a significantly improved animal model of wheat allergy because of the following two critical characteristics: (i) as opposed to our model described here that uses oral SSPE challenge to elicit anaphylactic responses, none of the previously described models had reported this method to elicit anaphylaxis to SSPE and (ii) as opposed to our model described here using detailed analysis of single-mouse data, we determined the correlations among the 4 quantitative readouts of wheat allergenicity (sIgE, tIgE, HSR, and MMCR), none of the previous models had reported such detailed analysis. Correlation analyses have established and validated the sIgE as a strong quantitative readout of wheat SSPE-allergic sensitization that leads to anaphylaxis upon oral challenge, and HSR and MMCR as quantitative readouts of sIgE-mediated allergic reactions. Establishing these characteristics was critical in a wheat food allergy animal model for the following reasons: (i) wheat allergies in humans typically occur through food ingestion. Therefore, simulating the mouse model to reflect human oral exposure conditions is vital to validating a model; (ii) validating the quantitative readouts of wheat allergenicity has major future applications. For example, one may use these readouts to determine whether the *in vivo* allergenicity of various wheat lines developed by breeding and cross-hybridization would be different, thereby, identifying potentially hypoallergenic and hyper-allergenic wheat. Furthermore, if genetically modified (GM) wheat is to be developed in the future, this model could be used to establish whether or not such GM wheat is “substantially equivalent” to non-GM wheat variety in eliciting clinical sensitization that leads to oral allergic reactions; validation of HSR and MMCR as markers of sIgE-mediated wheat allergy upon oral challenge can be used to establish this critical requirement in the assessment of the allergenic potential of GM foods as recommended by the FAO/WHO in their decision tree method (64, 65); (iii) one can use these readouts to examine the impact of food processing on wheat allergenicity, especially its effect on intrinsic sensitization and oral elicitation potencies; (iv) in addition, the readouts measured in our study may be useful to establish no-observed-adverse-effect level (NOAEL) and lowest-observed-adverse effect (LOAEL) for wheat. Until now, it has not been possible to do such research simply because validated quantitative markers of intrinsic sensitization and orally elicited wheat allergy were not available; and (v) this model can be used to elucidate the mechanisms of wheat food allergy.

Completion of model characterization in wheat allergy not only could help eliminate the hyper-allergenic wheat lines from the human food chain but also identify and develop hypo/non-allergenic wheat. Moreover, this model may be utilized for developing therapeutics to prevent/treat wheat allergies without the need to use excessive unnatural activation of the immune system using adjuvants.

To understand the immune mechanisms underlying wheat allergies, cytokine response has been studied in a limited number of animal models of wheat allergies. Using an adjuvant-based mouse model of gliadin allergy, Bodinier et al. (41) reported elevated IL-4 and IL-5 in the lung fluid of gliadin-allergic mice challenged by nasal route. Besides these prototypic Th2 cytokines, Jin et al. (47) reported elevation of Th1 and Th17 cytokines in mice that had developed atopic dermatitis upon chronic intraperitoneal exposures to durum wheat SSPE with alum-adjuvant. Later in 2020, Jin et al. (48) reported that Th1, Th2, and Th17 cytokines were increased in both an alum-adjuvant mouse model, as well as in an adjuvant-free mouse model upon intraperitoneal injections with durum wheat SSPE. Here, we further extend the knowledge underlying the cytokine mechanisms of wheat allergy by identification of selected Th1, Th2, and Th17 cytokines elevated in the spleen of skin-sensitized mice after nine skin exposures. Together, these studies demonstrate the critical role of these cytokines in wheat allergenicity irrespective of the specific protocol used and the target site organ used in the study (lungs, skin, and spleen). In all these conditions, wheat allergens consistently appear to activate pathogenic Th1, Th2, as well as Th17 biomarkers in the body. Therefore, these pathways may represent future potential therapeutic targets for wheat allergies. In addition, the selective elevation of IL-6 and a small panel of chemokines (CCL5, CCL20, CCL22, and CXCL1) in the spleen upon oral allergen but not saline challenge suggests their key role in eliciting anaphylaxis *via* the oral route. They may also represent therapeutic targets of anaphylaxis triggered upon wheat ingestion. Recent evidence indeed suggested that elevated IL-6 is a diagnostic marker of systemic anaphylaxis in human food allergy in general (66, 67). Thus, IL-6 and the chemokines identified in this model may be considered for investigation as potential diagnostic markers of anaphylaxis caused by wheat ingestion in humans.

We were surprised to note that in addition to Th2 cytokine activation, Th1 cytokines were also activated in this model. However, despite the activation of Th1 cytokines, we found robust IgE response, hypothermia shock response, and mucosal mast cell response. Therefore, we do not think that the Th1 activation will interfere with the investigation of allergy in this model.

We conducted a systematic individual mouse analysis to establish correlations among the four wheat allergenicity readouts in this model that has been rarely done before in any animal model of food allergy. The rationale for this was as follows: (i) sIgE measurements require extensive optimization for each food type, require higher volumes of blood samples, and data are difficult to compare across different laboratories (53, 54, 68). On the contrary, commercial kits are available for tIgE measurements; tIgE measurements are therefore easier and comparable across laboratories. Our data show that in this model, tIgE correlates with sIgE measurements, and therefore, could be used as a surrogate for sensitization analysis in case sIgE measurements are not feasible to do. However, modest to a poor correlations between tIgE with MMCR or HSR caution against using tIgE to predict oral allergic reactions in this model. In contrast, only sIgE responses appear to be strong predictors of oral anaphylaxis; (ii) hypothermic shock response (HSR) has been widely used because it is relatively cost-effective and easy to measure. However, it does not reveal the mechanism underlying the reaction. In contrast, MMCR as measured by MMCP-1 levels reveals that reactions are indeed mediated by the sIgE antibody interaction with the allergen on the mucosal mast cell surface in the gut (55). Therefore, a strong correlation between the MMCR and HSR in this model demonstrates that sIgE-mediated MMCR contributes to the HSR observed in this model and therefore provides a mechanistic basis for the anaphylaxis observed in this model; and (iii) using correlation analysis we determined the best time points for quantifying HSR that are most likely mediated by sIgE, and mucosal mast cells in this model. The time points that show the strongest correlations between sIgE vs. HSR and MMCR vs. HSR (i.e., 15–20 min post-oral challenge) are expected to be the best time points are recommended for future studies on oral anaphylaxis as they reflect HSR that is most likely associated with sIgE/mucosal mast cell degranulation response.

Wheat allergic mice in this study displayed clinical symptoms such as scratching and labored breathing upon oral challenge with SSPE. However, we did not observe diarrhea in these mice upon oral challenge with SSPE. Here, we wanted to develop objectively quantifiable readouts of wheat allergy. Therefore, we focused on hypothermic shock response and MMCP-1 measurements as quantitative readout of oral allergenicity as they are robust objective readouts of food allergenicity in mice in general.

In this study, we report long-term (eight exposures) skin-sensitization data. However, we have studied sensitization after short-term (four exposures) also. For example, significant specific IgE antibody responses appear after 4th skin exposure to wheat protein in this model (Supplementary Figure 2). Therefore, if the goal of a researcher were to study short-term sensitization, then just 4 exposures would be adequate. We noted that specific IgE levels continue to increase after 4 weeks of exposure and reach much higher levels after the 8th exposure. Our goal in this study was to induce severe life-threatening anaphylaxis in wheat allergic mice upon oral allergen challenge. Therefore, we sought to saturate the system with specific IgE levels that we were able to achieve by longer-term skin exposures as we demonstrate here. Our data show that life-threatening hypothermia shock responses are induced upon oral challenge. Therefore, if the goal of a researcher were to study life-threatening oral anaphylaxis with robust hypothermia shock responses, our data suggest that eight or more skin exposures would be used. Thus, depending on the goal, other researchers might choose the appropriate length of skin sensitization and customize this model to their individual needs. The dose of protein used for skin sensitization in this study was also based on previous optimization studies.

In human studies, it is common to orally challenge patients with whole wheat flour extract. In this mouse model study, we administered salt-soluble protein extract. This is because our goal of this study was to develop a life-threatening oral anaphylaxis mouse model of wheat allergy to salt-soluble wheat proteins. We accomplished this goal with an oral challenge dose of 20 mg of salt-soluble wheat protein per mouse. On a dry weight basis, whole wheat contains ~14% total protein (~11.6% would be of gluten and 2.6% would be salt-soluble non-glutens). Thus, to orally challenge mice with whole wheat extract to get optimal reactions to life-threatening anaphylaxis, we would have to challenge each mouse with ~0.84 g of whole wheat flour. The oral dosing volume advised for mice is generally 0.25–0.3 ml per mouse (assuming 25 g as the average body weight of mice). Having 0.84 g of wheat flour in 0.3 ml volume of saline would be technically not feasible to make, and orally administer in mice. Therefore, this is a technical limitation of the mouse model we have developed.

In summary, we report the development and characterization of a novel mouse-based *in vivo* tool for assessment of intrinsic sensitization without tape-stripping of stratum corneum or using adjuvant (that are commonly used in previous methods to create wheat-allergy models), followed by oral anaphylaxis to wheat. We also validate four quantifiable readouts of allergenicity that should be vital in future studies focused on: (i) identifying safer wheat lines; (ii) developing hypoallergenic wheat products using novel processing methods; and (iii) developing effective biologics and immunotherapies to prevent and treat wheat allergies. Finally, the panel of oral anaphylaxis-associated immune markers may represent diagnostic markers and/or therapeutic targets of life-threatening wheat anaphylaxis.

## Data Availability Statement

The original contributions presented in the study are included in the article/Supplementary Material, further inquiries can be directed to the corresponding author.

## Ethics Statement

The animal study was reviewed and approved by IACUC, Michigan State University, East Lansing, MI 48823.

## Author Contributions

Conceptualization and investigation: HG, VG, and PN. Methodology: HG, VG, PN, RJ, and RR. Validation and writing—review and editing: VG, HG, RR, and RJ. Formal analysis: VG, HG, and RJ. Resources, supervision, and funding acquisition: VG and PN. Data curation, writing—original draft preparation, and visualization: VG and HG. Project administration: VG. All authors have read and agreed to the published version of the manuscript.

## Funding

The United States Department of Agriculture (USDA)/National Institute of Food and Agriculture (NIFA); Hatch project MICL02486 (Accession Number: 1012322); Hatch project MICL01699; Agricultural and Food Research Initiative Competitive Program, grant number: 2018-67017-27876. RJ was supported by the John Harvey Kellogg Graduate Assistantship and the Academic Achievement Graduate Assistantship from the Michigan State University.

## Conflict of Interest

The authors declare that the research was conducted in the absence of any commercial or financial relationships that could be construed as a potential conflict of interest.

## Publisher's Note

All claims expressed in this article are solely those of the authors and do not necessarily represent those of their affiliated organizations, or those of the publisher, the editors and the reviewers. Any product that may be evaluated in this article, or claim that may be made by its manufacturer, is not guaranteed or endorsed by the publisher.
