# Supplementary material for: An Adjuvant-Free Mouse Model Using Skin Sensitization Without Tape-Stripping Followed by Oral Elicitation of Anaphylaxis: A Novel Pre-Clinical Tool for Testing Intrinsic Wheat Allergenicity
Source: Front Allergy. 2022 Jun 24;3:926576. doi: 10.3389/falgy.2022.926576 (PMC9552944; doi:10.3389/falgy.2022.926576)
Supplement: Supplementary file 1 [file Data_Sheet_1.pdf]

Gao et al (2022) Supplementary Figure 1.

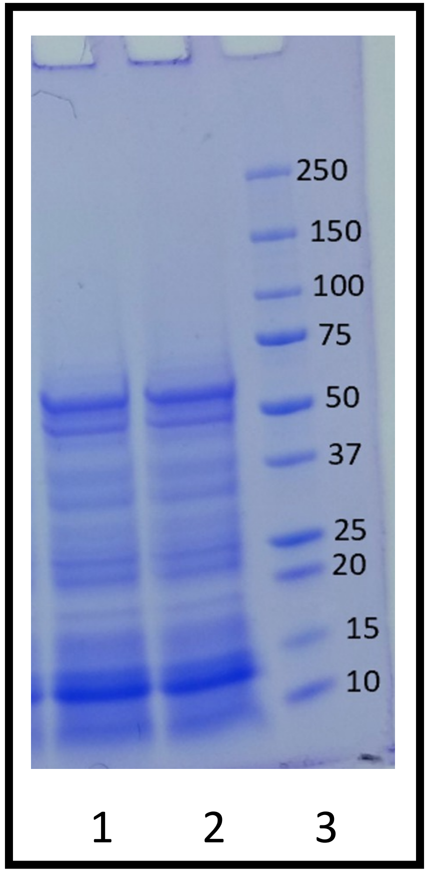

Sup. Fig. 1. Sodium Dodecyl Sulfate Polyacrylamide Gel Electrophoresis (SDS-PAGE) of salt-soluble protein extract (SSPE) from durum wheat used in this study. Lanes 1 & 2: SSPE from durum wheat (50 ug per lane in duplicate); Lane 3: molecular mass marker: 10-250 KDa.

Gao et al (2022) Supplementary Figure 2.

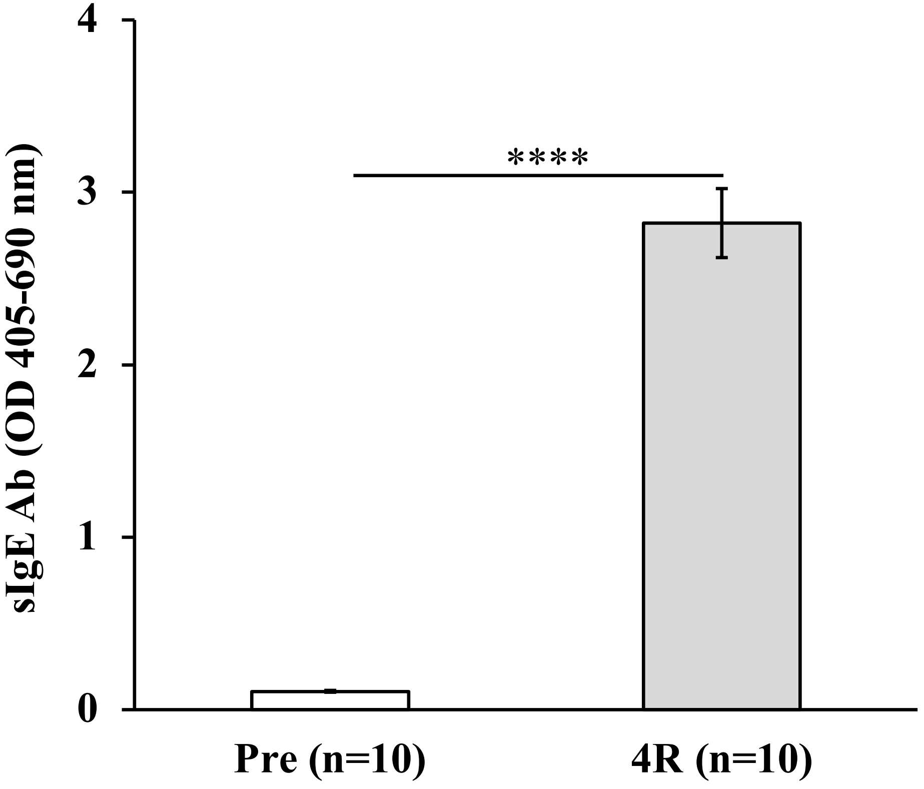

Gao et al (2022) Supplementary Figure 2.

Specific (s)IgE antibody response in Balb/c mice before and after four-skin exposures to salt-soluble protein extract (SSPE) from durum wheat. Blood was collected before 1st exposure (Pre) and after four-skin exposures (4R). Plasma was used in measurement of SSPE-specific IgE levels using an ELISA. **** Student's t test, $p < 0.001$; Ab: antibody.

Gao et al (2022) Supplementary Figure 3 A-B.

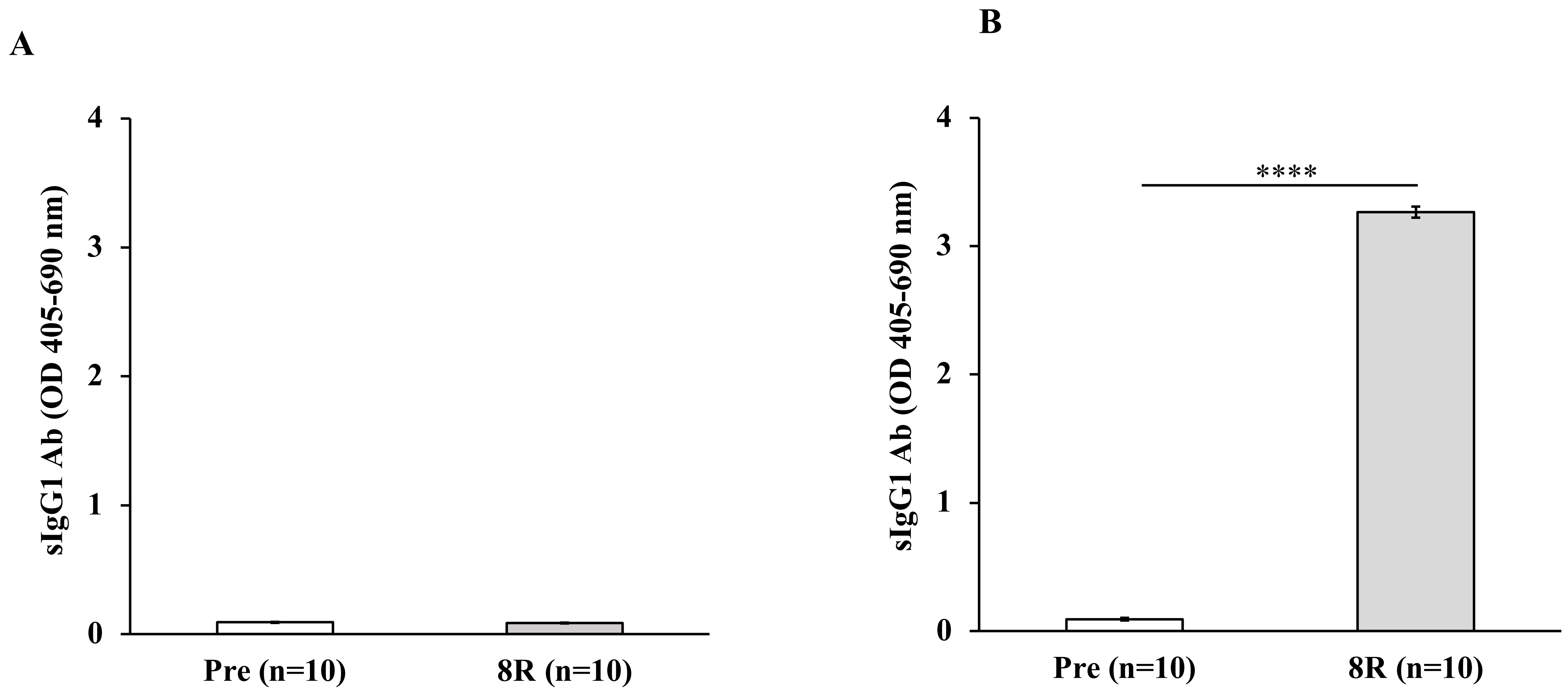

Gao et al (2022) Supplementary Figure 3 A-B.

Transdermal exposure to durum wheat salt-soluble protein extract (SSPE) elicited robust specific (s)IgG1 antibody responses in Balb/c mice. Mice were exposed to SSPE or to saline as described in Materials and Methods. Blood was collected before 1st exposure (Pre) and after 8th exposure (8R). Plasma was used in measurement of sIgG1 levels using an ELISA. Figure shows antibody levels in plasma with 1/40000 dilution. **(A) s**IgG1 levels in control mice. **(B)** sIgG1 levels in SSPE-sensitized mice. ****Student's t test, $p < 0.001$; Ab: antibody.
